# Socioeconomic, urban‐rural and sex‐based inequality in infant mortality rate: evidence from 2013 Yemen demographic and health survey

**DOI:** 10.1186/s13690-021-00589-1

**Published:** 2021-04-29

**Authors:** Betregiorgis Zegeye, Gebretsadik Shibre, Jemal Haidar, Gorems Lemma

**Affiliations:** 1 HaSET Maternal and Child Health Research Program, Shewarobit Field Office, Shewarobit, Ethiopia; 2 Department of Reproductive, Family and Population Health, School of Public Health, Addis Ababa University, Addis Ababa, Ethiopia; 3 Department of nutrition and dietetics, School of Public Health, Addis Ababa University, Addis Ababa, Ethiopia; 4 Chacha Health Center, Angolela Tera Health Office, Chacha, Ethiopia

**Keywords:** Infant mortality, Inequality, Yemen, DHS, Global health

## Abstract

**Background:**

The occurrence of Infant Mortality Rate (IMR) varied globally with most of the cases coming from developing countries including Yemen. The disparity in IMR in Yemen however, has not been well dealt and therefore we examined the IMR inequality based on the most reliable methodology in order to generate evidence-based information for some program initiatives in Yemen.

**Methods:**

Based on the World Health Organization (WHO) Health Equity Assessment Toolkit (HEAT) software, we analyzed the inequality across the different inequality dimensions in Yemen. The toolkit analyzes data stored in the WHO health equity monitor database. Simple and complex, and absolute and relative measures of inequality were calculated for the four dimensions of inequality (subpopulations) which included wealth, education, sex and residence. We computed a 95 % CI to assess statistical significance.

**Results:**

The analysis included 31, 743 infants. Absolute and relative wealth-driven, education, urban-rural and sex-based inequalities were found in IMR. Higher concentration of IMR was observed among infants from the poorest/poor households (ACI=-4.68, 95 % CI; -6.57, -2.79, R = 1.61, 95 % CI; 1.18, 2.03), rural residents (D = 15.07, 95 % CI; 8.04, 22.09, PAF=-23.57, 95 % CI; -25.47, -21.68), mothers who had no formal education (ACI=-2.16, 95 % CI; -3.79, -0.54) and had male infants (PAF= -3.66, 95 % CI; -4.86, -2.45).

**Conclusions:**

Higher concentration of IMR was observed among male infants from disadvantaged subpopulations such as poorest/poor, uneducated and rural residents. To eliminate the observed inequalities, interventions are needed to target the poorest/poor households, rural residents, mothers with no formal education and male infants.

## Background

Infant mortality is an important indicator of the overall physical health and socioeconomic condition of a community. High infant mortality rates (IMR) are generally indicative of unmet human health needs in basic health cares which include sanitation, medical care, nutrition and education in the first year of life [1, 2].

IMR has reduced globally over the past century though Sub-Saharan Africa (SSA) regions experienced an opposite trend, since the late 1990 s, due to the HIV pandemic [3] showing variations across countries. The highest rate is reported for SSA (75 infant deaths per 1000 live births) and the lower rate is reported in developed countries like Europe (11 infant deaths per 1000 live births [4]. The variation is even greater in developing than developed countries where IMR of 114 and 2 per 1000 live births is reported for Sierra Leone and Sweden, respectively indicating a 57-fold differences between the two countries [4]. When looked at IMR trends in Yemen, it was 75 in 1997, 69 in 2006 and 43 in 2013 showing marked decrement periodically [5] with significant reduction for urban than rural residents (36 vs. 51) [6].

It is apparent that the decline of child mortality rates is occurring differently world-wide among various countries and appears to be a challenge that the world is currently facing due to an increased inequality in child mortalities [7, 8]. According to the 2013 cross-country systematic review findings, socio-economic characteristics and social conditions are documented as the significant risk factors for IMR [9]. Variations in socio-economic status, health care and environmental aspect of different sub-populations affect the infant mortality directly [10]. Increasing in socioeconomic inequity like disparities in per capita national income, maternal educational status and access to health facilities generally lead to IMR inequalities in low and middle-income countries [11, 12]. Other important factors leading to variation in IMR are gender-based differences and residences [13, 14] though the assertion deserves an investigation in order to avail evidences on the extent of the problem in general and in particular to the study settings. [15]. Importantly understanding how IMR differs even within country is another basic issue that policymakers [16] need to know to use their limited resources soundly. In this regard, the first requirement for evidence-based policy to address health inequalities is to obtain evidences on health data that is stratified by subpopulation groups of the given country [17] and with this background information, we examined the magnitude of IMR by sociodemographic and economic status using different types of absolute and relative measures that combine both simple and complex measures in Yemen. The policy implication of the present study is to avail evidences for researchers as well as policy makers on the equity sensitive and responsive policy interventions from various dimensions [18].

## Methods

### Study area

The study was based on large data set stored in the WHO health equity monitor database for Yemen republic. Yemen is among the poorest country in the Middle East and North Africa [19], where the process of political transition has risen into a complete civil war since early 2015 [19, 20]. The war consequently led to internal disruption of services of all sectors among which healthcare services, displacement, causalities, and destruction of infrastructures such as main roads, bridges and airports [19, 20] are among the major ones. In the process, more than 12 million people experience food insecurity, 20.4 million of them deprived of safe water and adequate sanitation with more than 1.8 million children lost their school access. In addition, about 80 % or 21.1 of the people requiring humanitarian aids and protection [19, 20] particularly children since most are grappling with mass outbreaks of preventable diseases such as cholera, diphtheria, measles and dengue fever. Based on the projection evidences of previous data, IMR as of 2020 is estimated to be 43 per 1000 live birth [21, 22].

### Data source

We used a nationally representative data extracted from 2013 Yemen Demographic and Health Survey (YDHS), through the offline version of the WHO 2019 updated Health Equity Assessment Toolkit (HEAT) software (Available for free at https://www.who.int/data/gho/health-equity/heat-built-in-database--edition.) [23]. Although the detail discussion of the software is available elsewhere [23–25], the software is summarized as a toolkit which enables to scrutinize and analyze data on health inequalities within and between countries [26]. The software is tremendously valuable to explore the health disparity situation in a more systematic detail and comprises of the WHO Health Equity Monitor (HEM) database [26]. The database stores data coming from Demographic and Health Survey (DHS) and Multiple Indicator Cluster Survey (MICS) conducted in many low-or-middle income countries including Yemen. Currently, the database provides details of inequality assessment for more than 30 Reproductive, Maternal, Newborn and Child health indicators including IMR [24, 25].

The YDHS done in all the 20 governorates of the Republic including the capital city Sana’a with the aim to provide planners and decision makers with information on various health topics that includes IMR though in the survey, largest governorates were under sampled while the smallest ones were oversampled to produce sufficient samples for each governorate and the capital city.

The 2013 YDHS sample was selected using a stratified two-stage cluster design consisting of 800 clusters, with 213 in urban areas and 587 in rural areas [27]. The Birth Recode (BR file) was used for analysis of IMR [27].

### Variables and measurements

IMR is the variable of interest that we measured inequality of and refers to the probability (expressed as a rate per 1,000 live births) of a child exposed in a specific period dying before reaching their first birthday or the number of child death before celebrating first birth day per 1000 live births [28, 29]. The survey collected full birth histories for women aged 15–49 and years and children (including date of birth [28, 29]. A full birth history is a complete list of all children the woman has ever given birth which included date of birth, sex, survival status, age (if alive), and age at death (if died) [28, 29]. Birth histories captured all live births, including children who later died, but omitted stillbirths, miscarriages or abortions. Birth histories are collected in chronological order from first to last. Children born five years preceding the survey were included in the analysis [28, 29]. Inequality in IMR was measured in relation with four dimensions of inequality (sub-populations) that included economic status, education status, place of residence and sex (infant sex). Economic status was measured using a wealth index. The DHS constructs wealth index based on different household materials and durable assets collected during the survey. DHS uses Principal Component Analysis (PCA) to create the index and detail description on the creation of the wealth index is published elsewhere [30]. The wealth index was devided into five quintiles: poorest, poor, middle, rich and richest. Education refers to the highest level of schooling attained by the woman and is categorized as no education, primary school and secondary school and above, place of residents as rural vs. urban, child sex as male vs. female and subnational region included 20 region and one city (Ibb, Abyan, Sanaa City, Al-Baidha, Taiz, Al-Jawf, Hajjah, Al-Hodiedah, Hadramout, Dhamar, Shabwah, Sadah, Sana’a, Aden, Lahj, Mareb, Al-Mhweit, Al-Mhrah, Amran, Aldhalae and Reimah).

### Statistical analysis

We used the 2019 update WHO-HEAT software for data analysis and performed in steps. First, we disaggregated the IMR disparities by the above-mentioned five equity stratifiers (economic status, education status, infant sex, place of residence and subnational region). Then, we calculated summary measures; Difference (D), Ratio (R), Population Attributable Fraction (PAF) and Absolute Concentration Index (ACI) for each equity stratifiers. However, subnational region inequality in IMR was not calculated for all four summary measures either due to missing or unavailable data in two regions (Mareb and Al-Mhrah). Since ACI is applied only for ordered equity stratifiers such as economic and education status, it was not calculated for place of residence and sex. Details about calculation and interpretations of summary measures are described in the WHO health inequality handbook [18] and technical notes of HEAT software [23]. For some clarity on ACI, it is a complex, weighted measure of inequality that shows the health gradient such as IMR across multiple subgroups with a natural ordering, on an absolute scale and indicates the extent to which IMR was concentrated among the disadvantaged or the advantaged group. If there is no inequality in IMR, ACI takes the value zero. Positive values indicated a concentration of IMR among the advantaged subpopulation (richest and secondary school and above for economic and education status inequality dimensions, respectively), while negative values indicate a concentration of IMR among the disadvantaged subpopulation (poorest and no formal education for economic and education status inequality dimensions, respectively). ACI is usually negative for unfavorable health indicators such as IMR. The larger the absolute value of ACI, the higher the level of inequality of IMR.

Difference (D) is a simple, un-weighted measure of inequality that shows the absolute difference between two subgroups. If there was no inequality in IMR, D takes the value of zero. Greater (in absolute values) indicates higher levels of inequality in IMR. A positive value indicated a higher concentration of IMR among infants in the disadvantaged subpopulations (poorest, no formal education, rural residents and male for economic status, education status, place of residence and sex (infant sex) inequality dimensions respectively) and a negative value indicated that higher concentration of IMR among infants in the advantaged subpopulations (richest, secondary school and above, urban residents and female for economic status, education status, place of residence and sex (infant sex) inequality dimensions, respectively).

PAF is a complex, weighted measure of inequality that shows the potential for improvement in the national level of IMR, in relative terms, that could be reduced if all subgroups had the same level of IMR as a reference subgroup. PAF was calculated by dividing the population attributable risk (PAR) by the national average µ of IMR and multiplying the fraction by 100.

PAF takes negative values for adverse health outcome indicators like IMR. The larger the absolute value of PAF, the larger the degree of inequality. PAF is zero if no further reduction in IMR could be achieved, i.e. if all subgroups have reached the same level of IMR as the reference subgroup. The reference groups are subpopulation with lowest estimate of IMR; richest, secondary school and above, urban residents and female for economic status, education status, place of residence and sex (infant sex) inequality dimensions, respectively.

Ratio (R) is a simple, un-weighted measure of inequality that shows the relative inequality between two subgroups. If there is no inequality, R takes the value one. It takes only positive values and the further the value of R from 1, the higher the level of inequality.

### Ethical considerations

The demographic health surveys are available publicly and ethics approvals were completed by institutions that commissioned, funded, and managed the surveys. DHS surveys are approved by Inner City Fund (ICF) International and an in-country Institutional Review Board (IRB) to ensure protocols are in compliance with the U.S. Department of Health and Human Services regulations for the protection of human subjects. It is however worth noting that, in this study, we used publicly available DHS data which did not require further ethical clearance.

## Results

### Socio‐demographic characteristics of study participants

A total of 31, 743 sampled populations were included in the study. Of the total samples, 49 % were female infants. Nearly three-quarters (73.1 %) of the respondents were rural residents and 23 % of the respondents were from the poorest wealth index categories. Regarding educational status, 60.1 % of mothers had no formal education, while 29.3 % of mothers were completed their primary schools.

### Distribution of IMR across subpopulations

As shown in Table 1, the study shows that the national average of IMR in Yemen was 46.7 per 1000 live births. The magnitude of IMR among poorest was about 53 per 1000 live births (95 % CI; 46.06, 61.44) while for the richest categories was 33 per 1000 live births (95 % CI; 26.44, 41.20), showing marked disparities in terms of wealth index categories.

**Table 1:** Infant mortality rate disaggregated by the five equity stratifiers in Yemen: Evidence from 2013 Yemen demographic and health survey

Dimension of Inequality	Subgroup	Estimate (95 % CI)	Population
Economic status	Quintile 1 (poorest)	53.23(46.06, 61.44)	7313
	Quintile 2	58.26(50.18, 67.53)	6873
	Quintile 3	46.91(40.65, 54.07)	6356
	Quintile 4	37.45(31.71, 44.17)	5886
	Quintile 5 (richest)	33.03(26.44, 41.20)	5314
Education status	No education	49.74(45.23, 54.67)	19,075
	Primary school	45(39.63, 51.05)	9291
	Secondary school +	34.15(26.46, 43.97)	3377
Place of residence	Rural	50.79(46.50, 55.45)	23,210
	Urban	35.71(30.67, 41.56)	8533
Sex	Female	45.02(40.7, 49.78)	15,519
	Male	48.37(43.99, 53.15)	16,224
Subnational region	Ibb	51.50 (35.99–73.18)	3480
	Abyan	41.02 (28.27–59.16)	572
	Sanaa City	31.56 (24.07–41.29)	2586
	Al-Baidha	61.40 (53.56–70.31)	1253
	Taiz	50.88 (39.79–64.85)	3919
	Al-Jawf	26.07 (12.71–52.72)	277
	Hajjah	32.24 (23.15–44.73)	2152
	Al-Hodiedah	49.11 (38.98–61.71)	4037
	Hadramout	26.76 (18.98–37.62)	1566
	Dhamar	60.93 (49.66–74.55)	2644
	Shabwah	29.77 (19.71–44.72)	562
	Sadah	43.16 (31.05–59.72)	1046
	Sanaa	59.77 (47.68–74.68)	1720
	Aden	35.31 (22.67–54.60)	776
	Lahj	27.98 (18.49–42.13)	777
	Mareb	NA	237
	Al-Mhweit	54.97 (45.03–66.94)	1005
	Al-Mhrah	NA	121
	Amran	58.51 (46.16–73.92)	1353
	Aldhalae	42.61 (34.89–51.94)	804
	Reimah	53.42 (43.22–65.87)	847
National average		46.73	31,743

*Notes: NA data not available for the region*

In this study, slight differences in IMR was seen among infant who were born from mothers with no formal education (49.74, 95 % CI; 45.23, 54.67), and mothers who had attended secondary schools and above (34.15, 95 % CI; 26.46, 43.97). Similarly, higher magnitudes of IMR were seen among rural infants (50.79, 95 % CI; 46.50, 55.45) than urban (35.71, 95 % CI; 30.67, 41.56) (Table 1).

### Magnitude of inequalities in IMR

As presented in Table 2, we have seen that, absolute (ACI=-4.68, 95 % CI; -6.57, -2.79, D = 20.19, 95 % CI; 9.59, 30.78) and relative (PAF=-29.32, 95 % CI; -31.83, -26.82), ratio ( R = 1.61, 95 % CI; 1.18, 2.03) wealth-driven inequalities in IMR by all four summary measures with higher magnitude of IMR among infants were from disadvantaged subpopulations (poorest and poor).

In this study, absolute (ACI=-2.16, 95 % CI; -3.79, -0.54, D = 15.59, 95 % CI; 5.72, 25.45) and relative (PAF=-26.86, 95 % CI; -30.12, -23.59, R = 1.45, 95 % CI; 1.06, 1.85) education related inequalities in IMR were observed with higher burden among infants born from mothers who did not have formal education. The PAF measure further tells us, the 2013 national IMR could be reduced by about 27 % if the country would have avoided education related inequality.

Similarly, absolute (D = 15.07, 95 % CI; 8.04, 22.09) and relative (PAF=-23.57, 95 % CI; -25.47, -21.68, R = 1.42, 95 % CI; 1.17, 1.67) urban-rural inequality in IMR was observed with more concentration of IMR among infant of rural mothers.

There was no gender-based inequalities in IMR with simple measures (D, R). However, the PAF measure (complex measure) shows that relative sex-based inequality in IMR. PAF= -3.66, 95 % CI; -4.86, -2.45) (Table 2).

**Table 2:** Magnitude of inequalities in IMR using different summary measures in Yemen: Evidence from 2013 Yemen demographic and health survey

Dimensions of inequality	Summary measures	Estimate [95 % CI]
Economic status	ACI	-4.68 (-6.57, -2.79)
	D	20.19 (9.59, 30.78)
	PAF	-29.32 (-31.83, -26.82)
	R	1.61 (1.18, 2.03)
Educational status	ACI	-2.16 (-3.79, -0.54)
	D	15.59 (5.72, 25.45)
	PAF	-26.86 (-30.12, -23.59)
	R	1.45 (1.06, 1.85)
Place of residence	D	15.07 (8.04, 22.09)
	PAF	-23.57 (-25.47, -21.68)
	R	1.42 (1.17, 1.67)
Sex	D	3.34 (-3.08, 9.77)
	PAF	-3.66 (-4.86, -2.45)
	R	1.07 (0.92, 1.22)

## Discussion

The present study is the first comprehensive analysis of inequality regarding IMR in Yemen which made use of most reliable inequality analysis technique and shed light on how IMR differed greatly between different subpopulations. It is evident that we presented the coherence of the importance of the findings for setting priorities and focus on subpopulations who suffer most following the era of the Sustainable Development Goal (SDG) which advocates “leaving no one behind” principle [31]. Any systematic inequality between groups that is avoidable and unfair is inequity [32]. Therefore, IMR inequality that is not due to biological or natural difference is not just variation of IMR between subpopulations; it is rather inequity that should not have occurred. Interventions aiming to overcome IMR inequity between different subpopulations have to be underpinned by the paradigm of human right and egalitarian ethics in order to give it more weight to the problem.

It is apparent that in this study IMR are highly concentrated among poorest categories presumably due to health care services accessibility differences between the poor and rich. This assertion is supported by WHO which has stressed a positive link between wealth and better accessibility to medicine, “the first to benefit from the advances in medical technology and other health care improvements are those in the highest income classes; and merely slowly do the fruits of such progress filter down to the economically disadvantaged groups in the society” [33]. Individuals with higher economic status can get health care services in better equipped health facilities than poor who are often dependent on health insurance schemes that are provided by the government [34].

The male-female differentials of IMR have been supported by the complex measure, while both D and R did not show any differential clustering of the IMR between male and female. But when there is inequality, male children endure a higher burden of infant deaths and this finding corroborates with prior literature, where male children are reported to be at increased risk of dying before celebrating their first birth day than their female counterparts [13]. Child and infant death are higher for boys in virtually all nations due to mainly male infants have increased risk of birth complication and infectious diseases [35]. They are also more likely to be born prematurely [36] and are less physiologically mature at birth [37–41]. Evidence indicated that boys have higher risk of developing communicable diseases such as syphilis, malaria, tetanus, and diarrheal diseases [42], which partly explains the higher risk of death among boys than among girls. This is due to the fact that boys may have generally weaker immune system [43]. If male infant death had been reduced to a level in females, then the current national IMR would have been reduced by an approximately 4 % which is, a big drop that would meaningfully contribute to achievement of the “leaving no one behind” adage of the SDG. In contradiction, Chowdhury et al. (2017) in India showed huge survival advantage of male children in the post-neonatal period, where mortality among females was 35.0 % higher than mortality among male children [44] probably attributed to male sex preferences and this phenomenon makes India and Tonga as the outlier countries globally where girl infant death outnumbered boy’s [45].

The uneven distribution of infant death between male and female has huge policy implications; gender sensitive interventions need to be formulated in order to ensure both sexes are reached equally with appropriate equitable interventions.

Another important finding is that educational inequality of IMR was more pronounced among households with no education than those who had formal education. This is likely because educated mothers are conscious and practice improved birth spacing, have better awareness and make use of prenatal care and other health services [46] and earn higher income [47]. Similar explanation was forwarded by Vikram et al. where they mentioned maternal education to have impact on infant survival [48]. In the same manner, participation in higher education allows a woman to get a better occupation and hence a higher income/wealth level which again can have a desirable outcome on IMR [49]. Maternal education can also influence the attitudes of mothers towards traditional norms and beliefs including traditional infant caring practices, knowledge about illness and disease prevention practices, which have an impact on the infant and child survival [50]. Furthermore, educated mothers are likely to utilize modern health care facilities and are more aware about hygienic practices [51] in addition to taking care of themselves during pregnancy and provide the necessary care of their child through the most vulnerable stages of its life than non-educated mothers [50].

The other finding that emerged from our analysis is that rural settings perform poorly compared to urban areas. The IMR in the country would have fallen, on average, by a 24 % if the IMR in rural areas fell to a level in urban areas.

## Strengths and limitations

The study has few strengths. Firstly, we examined IMR inequality using the WHO HEM database by means of HEAT software, which permits to analyze inequality in high standard quality and comparable across countries. This is because of the re-analyzed DHS data is carried out by experts in health inequality. Secondly, analysis of inequality using absolute and relative as well as simple and complex summary measures could help policy makers and programmers to view the problem from different perspectives and plan interventions accordingly. The study however, had several limitations due to unavailability of data of earlier DHS in the HEAT software and we were unable to present over time trends. Other limitation is that the explanatory variables behind IMR inequalities are not identified and thus, further decomposition research in the future is recommended.

## Conclusions

The study shows absolute and relative socio-economic and urban-rural IMR inequalities with higher concentration among infants from the poorest households, uneducated mothers and rural residents. Moreover, relative sex-based inequality in IMR was seen with more concentration of IMR among male infants.

It is therefore essential for policy makers and other relevant stakeholders to design interventions by taking into account infants from disadvantaged subpopulations. In addition, more priority needs to be given during implementations of child survival interventions for male infants and those from rural residents by empowering of mothers through education and income generation schemes.

## Data Availability

The datasets generated and/or analyzed during the current study are available in the WHO’s HEAT version 3.1 [https://www.who.int/gho/health_equity/assessment_toolkit/en/].

## Abbreviations

ACI: Absolute Concentration Index
CI: Confidence Interval
D: Difference
DHS: Demographic and Health Survey
EA: Enumeration Area
HEAT: Health Equity Assessment Toolkit
ICF: Inner City Fund
IMR: Infant Mortality Rate
PAF: Population Attributable Risk
PCA: Principal Component Analysis
PSUs: Primary Sampling Units
R: Ratio
SDG: Sustainable Development Goal
WHO: World Health Organization
YDHS: Yemen Demographic and Health Survey
